# A Comprehensive Review on Asthma Challenges in Pregnancy: Exploring First Trimester Exacerbations and the Spectrum of Congenital Anomalies

**DOI:** 10.7759/cureus.49849

**Published:** 2023-12-02

**Authors:** Pallavi Yadav, Arpita Jaiswal, Archan Patel, Lucky srivani Reddy, Arman Sindhu

**Affiliations:** 1 Obstetrics and Gynecology, Jawaharlal Nehru Medical College, Datta Meghe Institute of Higher Education and Research, Wardha, IND; 2 Respiratory Medicine, Jawaharlal Nehru Medical College, Datta Meghe Institute of Higher Education and Research, Wardha, IND

**Keywords:** pregnancy health, comprehensive care, asthma management, maternal and fetal outcomes, first trimester exacerbations, asthma in pregnancy

## Abstract

This comprehensive review delves into the intricate relationship between asthma and pregnancy, specifically focusing on the challenges encountered in the first trimester and the ensuing impact on maternal and fetal health. Examining physiological changes during pregnancy reveals the dynamic interplay influencing respiratory function and immune responses. Key findings underscore the vulnerability to asthma exacerbations in the critical first trimester, emphasizing the potential risks to both maternal and fetal well-being. Maternal and fetal outcomes are discussed, emphasizing the associations between poorly controlled asthma and adverse perinatal outcomes. Implications for clinical practice highlight the importance of preconception care, continuous monitoring, and collaborative efforts between obstetricians and pulmonologists. Patient education emerges as a fundamental aspect to empower pregnant women in managing their condition. The conclusion emphasizes the imperative for comprehensive care, advocating for individualized treatment plans, multidisciplinary collaboration, and public health initiatives. By adopting this holistic approach, healthcare providers can navigate the complexities of asthma during pregnancy, ultimately ensuring the optimal health of both the expectant mother and her developing fetus.

## Introduction and background

Asthma, a chronic respiratory condition characterized by airway inflammation, poses unique challenges during pregnancy. As one of the most common pre-existing medical conditions in pregnant women, asthma demands special attention due to its potential impact on both maternal and fetal well-being. Understanding the intricacies of asthma in pregnancy is crucial for healthcare providers to deliver effective care tailored to the specific needs of this population [1].

Asthma is characterized by recurrent episodes of breathlessness, wheezing, chest tightness, and coughing, which may fluctuate in severity. In pregnancy, managing asthma becomes a delicate balance, as the physiological changes accompanying gestation can influence the course of the condition. These changes include alterations in respiratory function, immune responses, and hormonal levels, all contributing to the complexity of managing asthma during this critical period [2].

Pregnancy itself induces significant physiological changes, impacting the respiratory and immune systems. For women with asthma, these changes may exacerbate their existing condition, leading to increased risks for both the mother and the developing fetus. Poorly controlled asthma during pregnancy has been associated with adverse outcomes such as preterm birth, low birth weight, and increased perinatal mortality [1]. Beyond the immediate risks, addressing asthma challenges during pregnancy is vital for ensuring the long-term health of both the mother and the child. Emerging evidence suggests potential links between maternal asthma and the development of respiratory conditions in offspring, emphasizing the need for proactive management strategies [3].

The objective of this comprehensive review is to provide a nuanced exploration of the challenges posed by asthma during pregnancy, with a specific focus on exacerbations occurring in the first trimester and the potential spectrum of congenital anomalies. By synthesizing existing literature and examining current gaps in knowledge, this review aims to contribute to a better understanding of the complex interplay between asthma and pregnancy. This review will offer valuable insights into the optimal management of asthma during pregnancy through a thorough analysis of physiological changes, the impact of asthma medications, and the outcomes for both the mother and the fetus. Additionally, by highlighting areas that require further investigation, this review seeks to guide future research endeavours, ultimately advancing our ability to provide comprehensive and evidence-based care for pregnant women with asthma.

## Review

Physiology of pregnancy and asthma

Changes in Respiratory Function During Pregnancy

The respiratory system undergoes remarkable adaptations to meet the increased metabolic demands of pregnancy. As the gravid uterus expands, it elevates the diaphragm and alters thoracic compliance, leading to lung volume and capacity changes. While tidal volume increases, expiratory reserve volume and functional residual capacity decrease. These alterations may influence the perception of dyspnea in pregnant women and, when coupled with underlying asthma, can exacerbate respiratory symptoms [4]. The effects of pregnancy on airway resistance are multifaceted. Progesterone, a hormone elevated during pregnancy, acts as a respiratory stimulant, potentially ameliorating symptoms in some women. However, the physiological decrease in airway resistance may be less pronounced in asthmatic individuals, contributing to heightened bronchial responsiveness. Understanding these changes in respiratory function is pivotal for tailoring asthma management to the unique physiological milieu of pregnancy [2].

Impact of Pregnancy on Asthma Severity

Pregnancy's impact on asthma severity is variable, with women experiencing diverse outcomes. At the same time, approximately one-third of asthmatic women report improvement in symptoms; another third note worsening, particularly during the first trimester. The remaining third often observe stability in their asthma status. These fluctuations underscore the importance of individualized care plans that consider the dynamic nature of asthma during gestation [5]. Several factors contribute to the observed variations in asthma severity. Hormonal changes, immunological shifts, and alterations in lung mechanics collectively influence the course of the disease. Additionally, disparities in healthcare access, socioeconomic factors, and environmental triggers can further exacerbate or mitigate asthma symptoms during pregnancy. Recognizing these influences is crucial for optimizing asthma management and improving outcomes for both the mother and the fetus [6].

Hormonal Influences on Asthma Symptoms

Hormonal fluctuations, particularly increased levels of estrogen and progesterone, play a central role in modulating asthma symptoms during pregnancy. While progesterone's respiratory stimulatory effects can enhance bronchodilation, estrogen may contribute to airway inflammation and heightened bronchial reactivity. The intricate interplay between these hormones and their impact on asthma underscores the need for a nuanced understanding of the condition in pregnant women [7]. Moreover, the hormonal changes extend beyond the immediate gestational period, influencing the postpartum course of asthma. Research suggests that hormonal shifts post delivery can lead to a temporary worsening of asthma symptoms in some women. Recognizing these hormonal influences is pivotal for tailoring asthma management throughout the entire peripartum period, ensuring optimal respiratory health for the expectant mother and her newborn [8].

First trimester exacerbations

Understanding the First Trimester

The first trimester of pregnancy, encompassing weeks 1-12, is a critical period characterized by the establishment of embryonic development and organogenesis. This phase is marked by rapid cell division and differentiation, making it particularly sensitive to external influences. Understanding the unique challenges of asthma during this early gestational period is essential for mitigating potential risks to both the pregnant woman and the developing fetus [9]. During the first trimester, organ systems are forming, and any disruptions, such as asthma exacerbations, may have lasting consequences. The intricate interplay between the physiological changes of pregnancy and the underlying pathophysiology of asthma makes this trimester a crucial focal point for examining exacerbations and their potential impact [2].

Factors Contributing to Asthma Exacerbations in the First Trimester

Various factors contribute to the increased susceptibility of asthma exacerbations during the first trimester. Hormonal fluctuations, as discussed in the previous section, play a pivotal role, with increased progesterone potentially affecting bronchial smooth muscle tone. Additionally, immune function changes may influence asthmatic individuals' inflammatory response [1]. Environmental factors, including exposure to allergens and respiratory irritants, can exacerbate asthma symptoms, and pregnant women may encounter new challenges in avoiding such triggers. The altered pharmacokinetics of asthma medications during pregnancy may also contribute to fluctuations in disease control. Understanding these multifaceted contributors is essential for developing targeted interventions and preventive strategies tailored to the specific needs of pregnant women with asthma [10].

Effects of First Trimester Exacerbations on Maternal and Fetal Health

Asthma exacerbations during the first trimester of pregnancy can have significant effects on both maternal and fetal health. Research has shown that experiencing a severe exacerbation during the first trimester is associated with an increased risk of congenital malformations. A large representative cohort study found that having a severe exacerbation in the first trimester of pregnancy was associated with a significant odds ratio (OR) of 1.64 for any congenital malformation [11]. This highlights the importance of effectively managing asthma during pregnancy, particularly in the early stages, to minimize the risk of adverse outcomes for both the mother and the baby. Furthermore, excessive gestational weight gain (GWG) in the first trimester has been identified as a risk factor for asthma exacerbation during pregnancy. Studies have shown that the risk of exacerbation increases in a dose-dependent manner with excessive GWG in the first trimester [12]. This underscores the need for comprehensive prenatal care that includes monitoring and managing weight gain, particularly in women with asthma, to reduce the likelihood of exacerbations and their potential impact on maternal and fetal health.

In addition to the immediate effects on pregnancy, asthma exacerbations during pregnancy have been associated with poor offspring respiratory health, including asthma. It is crucial to address and manage asthma exacerbations during pregnancy to mitigate potential long-term effects on the respiratory health of the offspring [13]. Overall, the effects of first trimester exacerbations on maternal and fetal health underscore the importance of proactive asthma management during pregnancy. Healthcare providers should closely monitor and support pregnant women with asthma to minimize the risk of exacerbations and their potential impact on pregnancy outcomes [14].

Congenital anomalies and asthma medications

Overview of Common Asthma Medications

Asthma medications are commonly used during pregnancy to manage asthma symptoms and prevent exacerbations. However, there is some concern about the potential risk of congenital anomalies associated with exposure to these medications during the first trimester of pregnancy. Short-acting inhaled bronchodilators, such as albuterol and levalbuterol, and inhaled corticosteroids (ICS), such as budesonide, are recommended for women with persistent asthma. Oral corticosteroids, such as prednisone, may be necessary to treat severe asthma attacks, but are not preferred for regular asthma treatment during pregnancy [15]. Several studies have investigated the association between asthma medication use during pregnancy and the risk of congenital anomalies. A cohort linkage study found a small increased risk of congenital anomalies for women taking asthma medication during the first trimester of pregnancy [15]. Another study found that optimal management of asthma during pregnancy is imperative to reduce fetal risk of congenital anomalies associated with severe exacerbations [16]. However, it is essential to note that the increased risk of congenital anomalies for women taking asthma medication is small, with little confounding by maternal age or socioeconomic status [15].

In terms of specific asthma medications, a European case-malformed control study found nonsignificant OR of exposure to inhaled β2-agonists for spina bifida, cleft lip, anal atresia, and severe congenital heart defects in general [17]. ICS are generally considered the preferred asthma medication during pregnancy, as they are effective in controlling asthma symptoms and reducing the risk of exacerbations, with little to no increased risk of congenital anomalies [16]. Overall, while there is some evidence of a small increased risk of congenital anomalies associated with exposure to asthma medication during the first trimester of pregnancy, the benefits of effective asthma management during pregnancy generally outweigh the potential risks. Pregnant women with asthma should work closely with their healthcare providers to develop an individualized treatment plan that effectively manages their asthma symptoms while minimizing potential risks to the developing fetus [16].

Potential Risks and Safety Concerns of Asthma Medications in Pregnancy

Asthma medications are generally considered safe for use during pregnancy, and pregnant women with asthma need to continue their prescribed treatment to maintain control of their condition. Most asthma medications are inhaled, which allows them to go straight into the lungs, minimizing systemic absorption and reducing the potential impact on the developing fetus [18]. Pregnant women must keep their asthma under control throughout pregnancy, as uncontrolled asthma can lead to decreased oxygen in the blood, which may affect the baby's growth and increase the risk of premature birth and low birth weight [18].

Research has shown that the increased risk of congenital anomalies associated with asthma medication use during the first trimester of pregnancy is small, with little confounding by maternal age or socioeconomic status [15,16]. Additionally, optimal management of asthma during pregnancy is imperative to reduce the fetal risk of congenital anomalies associated with severe exacerbations [16]. Pregnant women with asthma should work closely with their healthcare providers to develop an individualized asthma action plan and review it regularly during pregnancy to ensure they are receiving the best asthma care [18]. Women need to discuss any concerns they have about their asthma medications with their healthcare team before making any changes, as abruptly stopping asthma medication can lead to uncontrolled asthma, which poses risks to both the mother and the baby [18].

Studies on the Association Between Asthma Medications and Congenital Anomalies

Several studies have investigated the association between asthma medication use during the first trimester of pregnancy and the risk of congenital anomalies [15]. This suggests that a small increased risk is not significantly influenced by factors such as maternal age or socioeconomic status. Optimal management of asthma during pregnancy has been highlighted as imperative to reduce the fetal risk of congenital anomalies associated with severe exacerbations [16].

This emphasizes the importance of effectively managing asthma during pregnancy to minimize the potential impact on fetal health regarding specific asthma medications. They have been shown to effectively control asthma symptoms and reduce the risk of exacerbations, with little to no increased risk of congenital anomalies [16]. This suggests that ICS may be safer for pregnant women with asthma. Overall, while there is evidence of a small increased risk of congenital anomalies associated with exposure to asthma medication during the first trimester of pregnancy, the benefits of effective asthma management during pregnancy generally outweigh the potential risks. Pregnant women with asthma should work closely with their healthcare providers to develop an individualized treatment plan that effectively manages their asthma symptoms while minimizing potential risks to the developing fetus [15,16].

Maternal and fetal outcomes

Impact of Asthma on Maternal Health During Pregnancy

Asthma's impact on maternal health during pregnancy extends beyond the immediate respiratory symptoms. Poorly controlled asthma has been associated with an increased risk of complications such as gestational hypertension, preeclampsia, and gestational diabetes. The inflammatory nature of asthma may contribute to systemic inflammation, influencing vascular function and metabolic pathways [10]. Exacerbations of asthma can lead to respiratory distress, limiting physical activity and potentially impacting maternal weight gain. The stress on the respiratory system during asthma episodes may also contribute to maternal anxiety and psychological distress. Understanding these broader implications is crucial for comprehensive antenatal care, ensuring respiratory health, and addressing the multifaceted aspects of maternal well-being [19].

Effects of Asthma on Fetal Growth and Development

Asthma's influence on fetal growth and development is a significant concern. Maternal asthma, particularly when poorly controlled, has been associated with an increased risk of preterm birth and low birth weight. These associations' complex mechanisms may involve altered placental function, maternal oxygen desaturation during exacerbations, and the inflammatory milieu affecting fetal development [20]. Moreover, fetal exposure to maternal inflammation during asthma exacerbations may impact the developing immune system, potentially contributing to an increased risk of respiratory conditions in childhood. Recognizing these potential effects on fetal growth and development is essential for developing targeted interventions to optimize outcomes for pregnant women and newborns [21].

Long-Term Consequences for Both Mother and Child

The consequences of asthma during pregnancy extend beyond the immediate perinatal period, influencing the long-term health of both the mother and the child. Studies suggest that maternal asthma may be associated with an increased risk of respiratory conditions in offspring, emphasizing the importance of effective asthma management during pregnancy [22]. For the mother, a history of asthma during pregnancy may have implications for her respiratory health in the postpartum period and later in life. Long-term follow-up studies are essential to understand the trajectory of asthma and related comorbidities in women with a history of asthma during pregnancy [2].

Management strategies

Preconception Asthma Management

Assessment of asthma control: The preconception phase sets the stage for optimal asthma management during pregnancy, beginning with a comprehensive assessment of asthma control. Healthcare providers thoroughly evaluate the frequency and severity of symptoms, lung function assessments, and an exploration of how asthma impacts daily activities. This holistic evaluation provides a baseline understanding of the patient's asthma status, laying the foundation for tailored preconception care strategies. The assessment enables healthcare providers to identify potential challenges and customize interventions that address the patient's specific needs, aiming to optimize asthma control before conception [23].

Medication review and adjustment: A critical component of preconception care involves meticulously reviewing and adjusting asthma medications. This process is designed to enhance symptom control while minimizing potential risks associated with specific medications during pregnancy. The focus is often on optimizing controller medications to achieve a balance between effective asthma management and mitigating potential risks to the developing fetus. This proactive approach to medication review ensures that the patient enters pregnancy with a well-adjusted treatment plan, fostering a more stable respiratory environment and reducing the likelihood of exacerbations during gestation [24].

Education and counselling: Providing comprehensive education and counselling during preconception is pivotal for actively empowering patients to manage their asthma throughout pregnancy. This educational initiative includes discussions on the importance of maintaining optimal asthma control, emphasizing the potential risks associated with uncontrolled asthma during gestation. Patient counselling fosters adherence to medication regimens, addresses any concerns or misconceptions, and recognizes early signs of exacerbations. By equipping patients with knowledge and strategies, healthcare providers enhance patient engagement and contribute to a proactive and informed approach to asthma management, setting the stage for a healthier pregnancy journey [25].

Monitoring and Adjusting Asthma Medications During Pregnancy

Regular asthma monitoring: Regular asthma monitoring involves a systematic approach to clinical assessments, including lung function tests and symptom reviews, designed to continuously evaluate and optimize asthma control during pregnancy. By implementing these assessments at regular intervals, healthcare providers can proactively identify changes in asthma status, enabling the prompt detection of exacerbations or deteriorating control. This vigilant monitoring strategy ensures that any necessary adjustments to the treatment plan can be initiated promptly, preventing the escalation of symptoms and mitigating potential risks to both the pregnant woman and the developing fetus. Regular asthma monitoring is integral to personalized care, providing a dynamic and responsive approach to managing asthma throughout pregnancy [26].

Pharmacological considerations: The careful consideration of pharmacological choices in asthma management during pregnancy is critical to ensuring maternal well-being and fetal safety. This involves a thorough evaluation of the safety profiles of asthma medications, with a focus on balancing the need for adequate asthma control against potential risks to the developing fetus. Medication adjustments may be necessary based on the evolving needs of the pregnant woman, taking into account physiological changes and the changing risk-benefit ratio associated with different medications. This deliberative approach to pharmacological considerations underscores the commitment to optimizing asthma management while prioritizing the safety of the pregnancy, requiring ongoing assessment and adjustment as needed [27].

Individualized treatment plans: Acknowledging the inherent variability in asthma presentations, developing individualized treatment plans is a cornerstone of effective asthma management during pregnancy. These plans are tailored to each patient's unique medical history, the severity of asthma, and individual responses to medications. By recognizing the diverse factors contributing to the complexity of asthma, healthcare providers can design treatment regimens that address specific needs and challenges pregnant women face. Individualized treatment plans ensure that the management approach is not a one-size-fits-all model but finely tuned to the nuances of each patient's condition, optimizing the potential for successful asthma control throughout the pregnancy journey [28].

Collaborative Care Approach: Involvement of Obstetricians and Pulmonologists

Joint care planning: The collaboration between obstetricians and pulmonologists in joint care planning represents a fundamental shift towards a unified approach to managing asthma during pregnancy. Through this collaborative effort, healthcare providers develop a comprehensive care plan that addresses both obstetric and respiratory aspects. This involves in-depth discussions on medication adjustments, ensuring that the treatment plan aligns with both the mother's respiratory needs and the safety of the developing fetus. Monitoring strategies are carefully tailored to accommodate the unique challenges posed by pregnancy, promoting a synchronized and proactive approach to asthma management. Plans for managing exacerbations are strategically devised, recognizing these events' dual impact on maternal and fetal well-being. Joint care planning creates a unified roadmap that optimally balances obstetrics and pulmonology health considerations [29].

Communication and information sharing: Effective communication and information sharing between obstetricians and pulmonologists form the backbone of a cohesive and integrated healthcare approach for pregnant women with asthma. Regular and open communication ensures that all aspects of care are considered, bridging the gap between obstetric care requirements and respiratory health needs. Obstetricians are kept informed about the intricacies of asthma management plans, allowing them to integrate this information seamlessly into overall pregnancy care. Simultaneously, pulmonologists stay updated on pregnancy-related developments, such as hormonal changes and obstetric milestones, ensuring that the respiratory care plan remains dynamically responsive to the evolving needs of both the mother and the fetus. This collaborative communication strategy enhances the overall quality and continuity of care [30].

Multidisciplinary consultation: In situations demanding specialized insights or interventions, a multidisciplinary consultation involving maternal-fetal medicine specialists, allergists, and neonatologists becomes a valuable component of comprehensive care. This approach ensures that diverse expertise contributes to the decision-making process, particularly in complex cases. Maternal-fetal medicine specialists offer insights into the obstetric implications of asthma, allergists bring specialized knowledge on environmental factors and triggers, and neonatologists contribute expertise on potential neonatal outcomes. This multidisciplinary consultation is instrumental in providing a holistic and nuanced understanding of the unique challenges presented by asthma during pregnancy, allowing for the development of a well-rounded and personalized care strategy [31].

Prevention and education

Importance of Asthma Education for Pregnant Women

Understanding asthma and pregnancy: Focused education on the intricate relationship between asthma and pregnancy is paramount for empowering pregnant women with asthma. This educational initiative aims to provide a clear comprehension of how hormonal changes, respiratory adaptations, and medication adjustments collectively influence asthma and overall health during pregnancy. By offering comprehensive insights into the physiological interplay, this education enables pregnant women to make informed decisions about asthma management, fostering a sense of agency over their health and well-being [27].

Medication adherence: Emphasizing the importance of consistent medication adherence is crucial in educating pregnant women with asthma. Clear and accessible communication regarding the safety of prescribed medications during pregnancy, coupled with insights into the potential consequences of uncontrolled asthma, serves to enhance medication compliance. This educational focus promotes the effective management of asthma symptoms. It addresses any concerns or misconceptions pregnant women may have about the safety and necessity of their prescribed medications during this crucial period [32].

Recognizing asthma triggers: Education geared towards recognizing and minimizing asthma triggers is essential for pregnant women managing asthma. This involves raising awareness about common triggers, including environmental factors like allergens and irritants, and imparting practical strategies to reduce exposure. By providing this knowledge, pregnant women can proactively manage their surroundings, mitigating the risk of exacerbations. This aspect of education empowers women to create a supportive environment conducive to optimal respiratory health throughout their pregnancy [33].

Lifestyle Modifications for Asthmatic Pregnant Women

Smoking cessation: Addressing smoking as a significant risk factor, interventions for smoking cessation are imperative for pregnant women with asthma. Smoking not only exacerbates asthma symptoms but also poses severe risks to both maternal and fetal health. Smoking cessation programs tailored to pregnant women with asthma should be readily accessible, providing support, counselling, and resources to facilitate a tobacco-free lifestyle. These programs play a crucial role in mitigating the adverse effects of smoking, promoting healthier pregnancies, and reducing the overall burden on respiratory health for both the expectant mother and the developing fetus [34].

Allergen and irritant avoidance: Comprehensive guidance on minimizing exposure to common allergens and irritants is vital for managing asthma during pregnancy. This involves practical recommendations for maintaining a clean and allergen-free living environment, including strategies for reducing exposure to pet dander, dust mites, and mold. In occupational settings, pregnant women with asthma should receive advice on protective measures to minimize exposure to workplace irritants. These proactive measures contribute to symptom control, reduce the likelihood of exacerbations, and create a supportive environment for optimal respiratory health during pregnancy [35].

Regular exercise and healthy diet: Encouraging a lifestyle that includes regular, moderate exercise and a balanced, nutritious diet benefits overall well-being during pregnancy, including women with asthma. Exercise can contribute to improved cardiovascular health and may aid in asthma management. However, exercise plans must be individualized and discussed with healthcare providers to ensure they align with each pregnant woman's needs and limitations. Similarly, promoting a healthy diet supports general health and may positively influence asthma outcomes. This holistic approach underscores the importance of lifestyle modifications tailored to the unique circumstances of pregnant women with asthma, promoting a comprehensive strategy for managing respiratory and overall health [36].

Public Health Initiatives for Asthma Awareness in Pregnancy

Educational campaigns: Educational campaigns are pivotal in raising awareness about pregnancy asthma among the general population and expectant mothers. Through diverse channels such as social media, pamphlets, and healthcare facilities, these campaigns aim to disseminate accurate and accessible information. They debunk common misconceptions surrounding asthma and pregnancy, fostering a better understanding of the condition's nuances. Emphasizing the significance of early detection and proactive management, these campaigns improve health outcomes by empowering pregnant women to make informed decisions about their asthma care [37].

Professional training programs: Targeted training programs for healthcare professionals are essential in equipping them with the specialized knowledge required to navigate the unique challenges presented by asthma during pregnancy. Obstetricians, pulmonologists, and primary care providers benefit from comprehensive training that delves into the physiological intricacies, medication considerations, and collaborative care aspects specific to pregnant women with asthma. By enhancing healthcare professionals' understanding of these complexities, training programs contribute to improved identification of asthma-related issues, more effective management strategies, and enhanced care coordination, ultimately ensuring better outcomes for pregnant women and their unborn children [38].

Community support programs: Establishing community support programs creates a network to address the social and emotional dimensions of managing asthma during pregnancy. These programs provide a valuable resource for pregnant women with asthma, offering a platform for shared experiences and peer support. By fostering a sense of community and empowerment, these programs contribute to mental well-being and resilience. Additionally, community support programs can offer practical resources, educational materials, and guidance, creating a holistic support system beyond the clinical setting. This community-based approach recognizes the importance of social connections in promoting overall health and well-being during pregnancy for women managing asthma [39].

## Conclusions

This comprehensive review has illuminated the intricate dynamics of asthma during pregnancy, particularly emphasizing the challenges encountered in the first trimester and the subsequent implications for maternal and fetal health. Noteworthy findings underscore the significant physiological changes during pregnancy, the heightened vulnerability to exacerbations in the initial stages, and the potential long-term consequences for both mother and child. From a clinical perspective, these insights emphasize the importance of preconception care, continuous monitoring, and collaborative efforts between obstetricians and pulmonologists. The crucial role of patient education in fostering adherence to treatment plans and lifestyle modifications is paramount. Ultimately, the need for comprehensive care emerges as a central theme, emphasizing the importance of individualized treatment, multidisciplinary collaboration, and public health initiatives to enhance awareness. By embracing this holistic approach, healthcare providers can navigate the nuanced intersection of asthma and pregnancy, ensuring optimal outcomes for expectant mothers and their newborns throughout the entire peripartum continuum.
